# Multigenerational Effects of Heavy Metals on Feeding, Growth, Initial Reproduction and Antioxidants in *Caenorhabditis elegans*

**DOI:** 10.1371/journal.pone.0154529

**Published:** 2016-04-26

**Authors:** ZhenYang Yu, Jing Zhang, DaQiang Yin

**Affiliations:** 1 Key Laboratory of Yangtze River Water Environment, Ministry of Education, College of Environmental Science and Engineering, Tongji University, Shanghai 200092, P. R. China; 2 College of Biological, Chemical Sciences and Engineering, Jiaxing University, Jiaxing, Zhejiang 314001, P. R. China; East Carolina University, UNITED STATES

## Abstract

Earlier studies showed that toxicities of excessive metals lasted over generations. Yet, these studies mainly employed one-generation exposure, and the effects of multigenerational challenges need further studies. Presently, *Caenorhabditis elegans* were exposed to cadmium, copper, lead and zinc for four consecutive generations (G1 to G4) at environmental concentrations. The feeding, growth, initial reproduction, superoxide dismutase (SOD) and catalase (CAT) were determined. All data were represented in the percentage of that in control (POC), and POC in the control was normalized to 100%. In G1 and G2, the POC values in feeding, growth and initial reproduction were generally within 10% of the control (100%), indicating non-significant effects. The POC values in SOD and CAT were significantly higher than 100%, showing stimulatory effects. In G3 and G4, the POC values in feeding, growth and initial reproduction were significantly lower than 100%, showing inhibitory effects which were more severe in G4 than in G3. Meanwhile, SOD and CAT continuously showed stimulatory effects, and the stimulatory effects on SOD increased from G1 to G4. The effects with multigenerational challenges were different from those in one-generation exposure. The effects in later generations demonstrated the importance of multigenerational challenges in judging long-term influences of metals.

## Introduction

Excessive metals were recently reported to have long-term environmental effects with influences across generations. For example, the growth of daughters (marked as G2) of *Caenorhabditis elegans* was inhibited more than that of parents (G1) which were exposed to cadmium (Cd), copper (Cu), lead (Pb) and zinc (Zn) [1]. Initial reproduction in G3 of *C*. *elegans* significantly decreased compared with that in G1 that was subjected to gold nanoparticles [2]. The immune responses remained apparent in G2 of *Protophormia terraenovae*, even though the Cu exposure occurred in G1 [3]. However, most studies employed one-time exposure in the parent generation (G1), and did not consider exposure in subsequent generations, *i*.*e*., multigenerational challenges.

For now, only a few studies considered such multigenerational challenges in studying environmental effects of metals. For example, mercuric chloride inhibited the reproduction of *Tigriopus japonicas*, and the inhibitory effects were more severe after three exposure generations (G1 to G3) [4]. Cadmium increased the tolerance of *Spodoptera exigua* larvae after 33 exposure generations with lower mortality and stronger total antioxidant capacity [5]. The results of multigenerational exposure showed significant differences from those of one-generation exposure [5]. Therefore, effects with multigenerational challenges should be paid more attention to for a thorough evaluation on long-term effects of pollutants.

The present study explored the long-term effects of metals on *C*. *elegans* with multigenerational challenges. The choice of *C*. *elegans* (a free-living nematode) was due to the advantages of its easy handling in the exposure set-up [1, 2, 6, 7], and also its multiple available indicators. For example, the nematode feeding, growth and reproduction have been widely used in studying metal effects [1, 8–12]. Moreover, the nematode antioxidants, e.g., superoxide dismutase (SOD) and catalase (CAT), were inducible to scavenge reactive oxygen species generated by metals [11]. Such antioxidant responses provided essential biochemical information to explain the observed apical effects [13].

In the present study, *C*. *elegans* were exposed to Cd, Cu, Pb and Zn for four consecutive generations from G1 to G4. Results showed that, in G1 and G2, the feeding, growth and initial reproduction were not significantly influenced, while SOD and CAT were stimulated. Effects in G1 and G2 were generally weaker than those in earlier one-generation exposure studies. In G3 and G4, the feeding, growth and initial reproduction showed significant inhibitory effects, and the SOD stimulations increased. Effects in G3 and G4 demonstrated that some biological changes caused by pollutants may be not observed until second challenges in later generations. Effects in multigenerational challenges should be considered carefully in judging long-term influences of metals.

## Materials and Method

### Tested chemicals

Stock solutions of CuCl_2_, Pb(NO_3_)_2_, Cd(NO_3_)_2_·4H_2_O and ZnCl_2_ were prepared with sterilized K-medium (0.051 M NaCl and 0.032 M KCl) [1, 14]. Two exposure concentrations were set up with 0.1 mg L^-1^ as the lower one, and 10 mg L^-1^ as the higher one. The exposure concentrations were selected to avoid severe worm toxicity according to the median effective concentrations (EC_50_) [8, 15]. Such concentrations also represented environmental levels of the metals [16].

### Preparation of nematode

*C*. *elegans* and its food *E*. *coli* OP50 were provided by Department of Biochemistry and Molecular Biology, Southeast University Medical School, Nanjing, China. Briefly, *E*. *coli* OP50 was cultured in sterile lysogeny broth (LB) culture medium, and *C*. *elegans* was cultured on solid nematode growth medium (NGM) plates [17]. Gravid nematodes were bleached for 5–10 min at room temperature in fresh clorox solutions containing 0.5 M NaOH and 1% NaOCl (diluted from antiformin, 4–6%, Sinopharm Group Co. Ltd., China) [17]. Then, the age-synchronized eggs were washed in sterile K-medium before their use in experiments.

### Exposure

The exposure was carried out in liquid medium according to earlier studies [1, 18, 19]. Briefly, the aqueous exposure was performed in 24-well sterile plates (with cover, Corning, Inc., USA). There were one control (sterilized K-medium) and two concentrations (0.1 and 10.0 mg L^-1^). Each concentration and the control used one whole plate (24 wells as 24 replicates). Each well contained 500 μL metal solution or K-medium (control), 200 μL K-medium containing approximately 50 nematode eggs, and 300 μL bacteria suspensions (*i*.*e*., 1000 μL in each well). Sterilized water (500 μL) was added in the interspaces between wells to keep the humidity and decrease the water evaporation during the exposure period.

In respect of nematode eggs, the eggs in 20 μL K-medium (abbreviated as N_20_) were counted under a microscope with three independent trials, and the number was multiplied by 10 to indicate the total egg number in 200 μL (abbreviated as N_200_). If the average N_20_ was more than 6 or less than 4 (*i*.*e*., N_200_ > 60 or N_200_ < 40), more K-medium was added into or some supernatant K-medium was drawn out of centrifuge tubes, until the average N_20_ was approximately 5 (*i*.*e*., N_200_ ≈ 50). The nematode number choice was based on both the influence of crowded population and the experiment arrangement in earlier studies [20].

In respect of the bacteria, they were first collected from LB culture medium by centrifugation at 4000 rpm for 5 min. Next, they were re-suspended with K-medium to make a bacterial suspension. Then, 300 μL bacterial suspension was added 700 μL K-medium to test optical density at 570 nm (OD_570_) with three independent trials. If the average OD_570_ was more than 1.0 or less than 0.8, more K-medium was added into or some supernatant K-medium was drawn out of the bacterial suspension, until the OD_570_ was approximately 0.9. At last, the bacterial suspension was equilibrated (1:1, V/V) with the metal or control solutions for 24 h, after which the bacteria were ready for nematode exposure. The treatment on bacteria resulted from a consideration on the metal adsorption into bacteria and the contribution of dietary exposure [11]. The amount of bacteria was enough to support 50 nematodes to reach full growth which was indicated by the appearance of embryos and vulva.

Based on preliminary experiments, the exposure duration was 5 days, which ensured the nematode reproduction capacity, and also enabled the separation of parent and newborn nematodes. On the 5^th^ day, approximately 25 parent nematodes in each well of any concentration group or control were collected to measure the effects on the first generation (G1). The remaining nematode in each concentration group or control (25 parent nematodes in each of 24 wells) were collected and bleached to gain the nematode eggs (G2) that were used in the second generation exposure with the same procedure as their parents experienced. On the 5^th^ day of G2 exposure, nematodes were measured for the effects in G2, and also bleached to gain the eggs (G3) that were used in the third generation exposure. The exposure was carried on until there were not enough eggs (< 8 wells for each concentration) for the succeeding exposure.

### Feeding measurement

The feeding abilities of *C*. *elegans* were expressed by determining the declining of food, based on the changes of optical density of the bacteria at 570 nm (ΔOD_570_). Such method was widely employed in studying toxic effects of metals [11, 15]. On the 1^st^ day of each exposure generation, OD_570_ of each well was recorded immediately (OD_570, beginning_) after metal (or control) solutions, bacteria and eggs were mixed. On the 3^rd^ day of each generation, the OD_570_ of each well was recorded again (OD_570, ending_), and the percentage change of OD_570_ (ΔOD_570, with nematodes_ = (OD_570, beginning_−OD_570, ending_)/OD_570, beginning_) was calculated and presented as ΔOD_570, with nematodes_. At the same time, the OD_570_ values of 24-well plates that contained the same bacteria and metal (or control) solutions but without nematodes were also measured to calculate the bacterial growth which was presented as ΔOD_570, without nematodes_. Then, the difference between the ΔOD_570_ in wells with and without nematodes was used to indicate the feeding ability of nematodes in the well (*i*.*e*., feeding ability = ΔOD_570, with nematodes_−ΔOD_570, without nematodes_).

### Growth measurement

The growth of nematodes was indicated by the body length which was measured on the 3^rd^ day of each generation in the same way as previously described [11, 21]. Briefly, nematodes were transferred onto clean NGM plates without food after a wash with sterilized distilled water. After 2 h allowing water evaporate, a dissecting microscope was used to capture the images of nematodes. A polyline was drawn following the nematode’s midline from the head to the tail and back to the head. The body length of a nematode was calculated as half length of the polyline. At least 15 nematodes were measured for each treatment in each generation.

### Reproduction measurement

In natural environment, animals have a strategy to reproduce as fast and as much as possible before accident death. Therefore, any decrease or delay of the initial reproduction implies that the animal has reduced chances of maintaining a viable population in nature [22]. Accordingly, the initial reproduction, *i*.*e*., the accumulated number of offspring (offspring and eggs) within 72 h since synchronization, was measured as an early warning indicator to demonstrate the multigenerational effects of heavy metals on the nematode reproduction. Briefly, on the 3^rd^ day of each generation’s exposure, 20 μL evenly stirred exposure mixture were drawn out from each well and dropped in the center of a glass slide, and then the nematodes were counted under a microscope. Parent nematodes and the offspring (including eggs) were distinguished by size. The counting was repeated at least twice on replicated glass slides. The initial reproduction was calculated as the number of offspring and eggs divided by the number of parents.

### Superoxide dismutase and catalase assay

Antioxidant protein abundance and activities were well correlated with each other [23]. In the present study, the protein abundance of SOD and CAT were measured to indicate the antioxidant activities according to the procedure in our previous study [11]. Briefly, nematodes were collected into centrifuge tubes. After a 30 min-settlement, the pellets were transferred into new centrifuge tubes and washed with ice-cold phosphate buffered saline (PBS, pH 7.0). Next, the tubes were centrifuged at 5000☓g for 5 min (4°C), and the pellets were stored at -26°C overnight. Then, the pellets were homogenized with pestles in ice bath. After a centrifugation at 5000☓g for 5 min (4°C), the supernatants were aliquoted for subsequent determination. Both SOD and CAT were determined using the enzyme-linked immune-sorbent assay (ELISA) kits (R&D Systems, Inc., USA). The total protein (TP) in each sample was also measured by ELISA kits, and SOD and CAT were expressed as their proportions (P) in TP to eliminate the differences of nematode numbers among samples.

### Data presentation and statistical analysis

The data presentation and statistical analysis were performed according to earlier reports [24]. First, the values of each indicator in the control with 24 replicates were averaged. Next, every value of the indicators in the 24 replicates from each group (including the control) was calculated as a percentage of the average value in the control (percentage of control, POC). Then, the 24 POC values in each group were used to calculate the means and standard errors. The values in the control were normalized to 100%. The values in the exposure group lower than 100% indicate inhibitory effects and those higher than 100% indicate stimulatory effects. The 24 POC values in each group were used in ANOVA with Tukey’s test as the post hoc analysis to find statistical differences (p < 0.05) between each group and the control (* in figures), between concentrations in the same generation (+ in figures), and between generations at the same concentration (# in figures). The ANOVA was also performed to find the difference between metals at the same concentration. Pearson’s correlation was carried out to seek connections between indicators. Both ANOVA and Pearson’s correlation were performed in Origin Pro 8.5 (Origin Lab Corp., USA).

## Results

### Multigenerational effects of metals on nematode feeding

**Fig 1:**
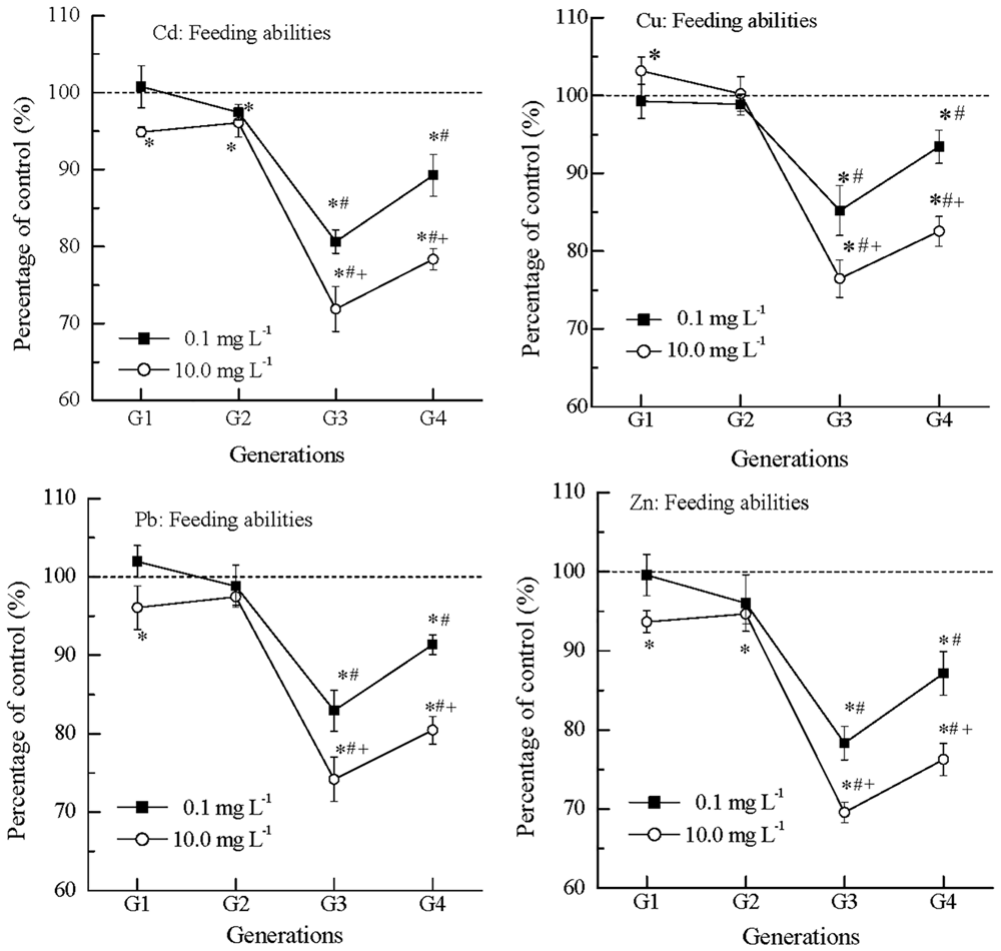
Feeding ability of *C*. *elegans* showed fluctuation in inhibition over generations from G1 to G4 in exposure to Cd, Cu, Pb and Zn. The ΔOD_570_ percentage change in the control was 55.3% ±1.7%, and the average value was normalized to 100% (dash line); *: significantly different from the concurrent control, p < 0.05; #: significantly from the earlier generation, p < 0.05; +: significantly different from the lower concentration, p < 0.05; Error bar: standard error.

The effects of metals on feeding abilities of *C*. *elegans* are shown in Fig 1. In the first generation (G1), metals at 0.1 mg L^-1^ did not significantly influence the feeding compared with the control. At 10.0 mg L^-1^, the POC values of cadmium (Cd), lead (Pb) and zinc (Zn) were 94.9%, 96.1% and 93.7% (inhibitory effects), respectively, while the POC value of copper (Cu) was 104.2% (stimulatory effects). In the second generation (G2), the POC values were closer to 100% than those in G1, indicating lower inhibition or stimulation.

In the third generation (G3), the POC values at 0.1 mg L^-1^ were 80.6%, 85.2%, 82.9% and 78.3% in Cd, Cu, Pb and Zn, respectively. The POCs at 10.0 mg L^-1^ were 71.9%, 76.5%, 74.2% and 69.6% in Cd, Cu, Pb and Zn, respectively. Such POC values indicated that the inhibitions in G3 were more severe than those in G1. In the fourth generation (G4), the POC values were 89.2%, 93.4%, 91.3% and 87.1% at 0.1 mg L^-1^, and 78.4%, 82.6%, 80.4% and 76.3% at 10.0 mg L^-1^ in Cd, Cu, Pb and Zn, respectively. The inhibitions in G4 were less than those in G3.

### Multigenerational effects of metals on nematode growth

**Fig 2:**
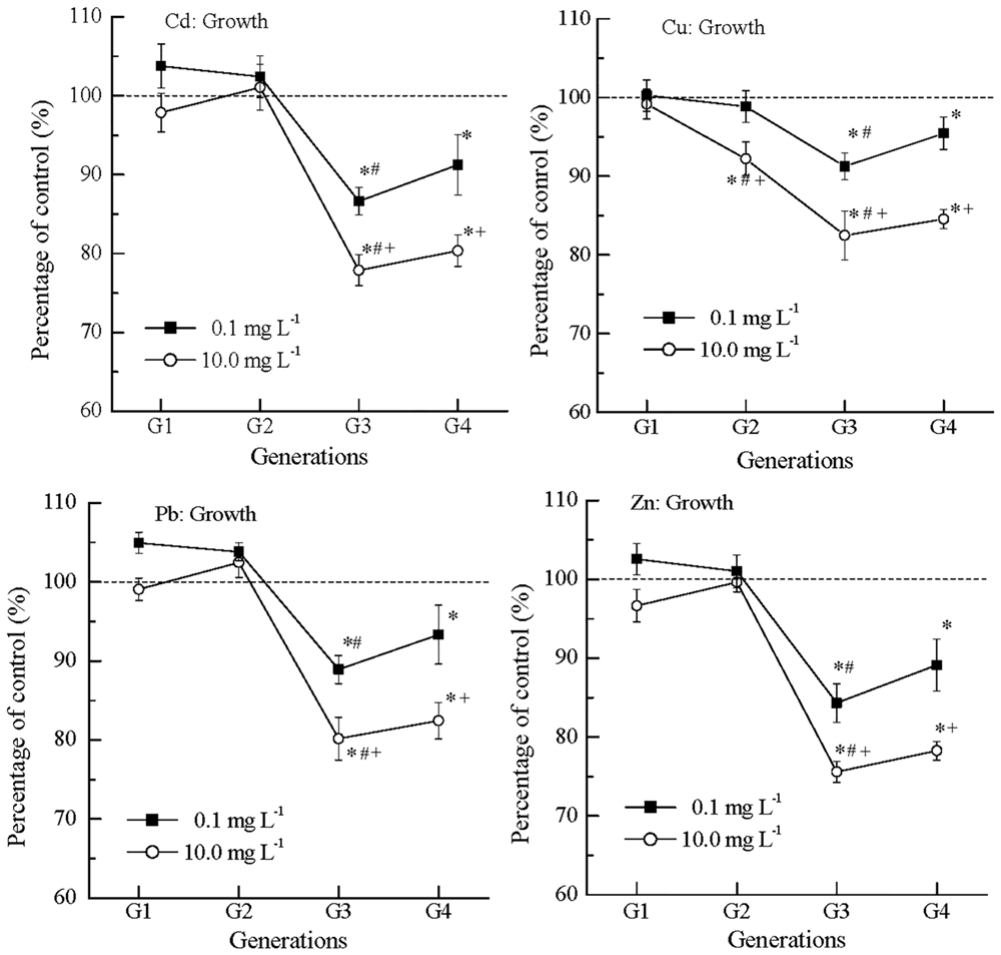
The nematode growth showed fluctuation in inhibition over generations from G1 to G4 in exposure to Cd, Cu, Pb and Zn. The body length of nematodes in the control was 1.14 ± 0.11 mm, and the average value was normalized to 100% (dash line); *: significantly different from the concurrent control, p < 0.05; #: significantly from the earlier generation, p < 0.05; +: significantly different from the lower concentration, p < 0.05; Error bar: standard error.

The multigenerational effects of metals on nematode growth are shown in Fig 2. In G1 and G2, the POC values were generally within 10% of the control, showing slight inhibitory or stimulatory effects that were observed in effects on feeding. In G3 and G4 (Fig 2), the POC values of Cd ranged from 86.6% to 91.2% at 0.1 mg L^-1^, and from 77.9% to 80.4% at 10.0 mg L^-1^, indicating similar inhibitory effects in G3 and G4. Such similarity was also observed in Cu (91.2% to 95.4% at 0.1 mg L^-1^ and 82.5% to 84.6% at 10.0 mg L^-1^), Pb (88.9% to 93.3% at 0.1 mg L^-1^ and 80.2% to 82.5% at 10.0 mg L^-1^) and Zn (84.3% to 89.1% at 0.1 mg L^-1^ and 75.6% to 78.3% at 10.0 mg L^-1^). The inhibitions of metals in G3 and G4 were significantly more severe than those in G1 and G2. The effects on growth were similar to those of feeding (Fig 1). The similarity was verified by high values in Pearson’s correlation coefficient (Pearson’s r greater than 0.97, S1 Table).

### Multigenerational effects of metals on nematode reproduction

**Fig 3:**
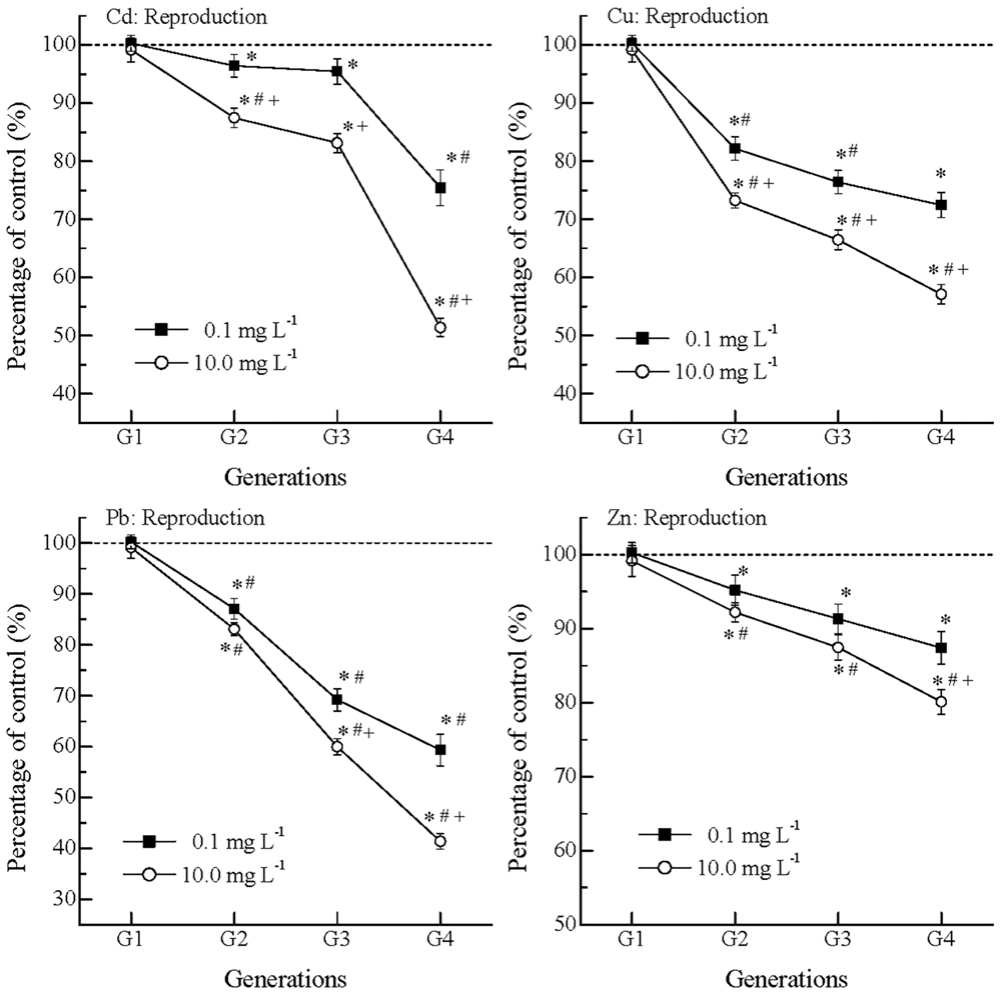
The nematode initial reproduction decreased over generations from G1 to G4 in exposure to Cd, Cu, Pb and Zn. The offspring number in the control was 49 ± 6 within 72 h, and the average value was normalized to 100% (dash line); *: significantly different from the concurrent control, p < 0.05; #: significantly from the earlier generation, p < 0.05; +: significantly different from the lower concentration, p < 0.05; Error bar: standard error.

The multigenerational effects of metals on the nematode initial reproduction are shown in Fig 3. In G1, the POC values of metals were around 100% without significant differences from the control. In G2 and G3, the POC values of Cd were 96.4% and 95.4% at 0.1 mg L^-1^, and they were 87.5% to 83.1% at 10.0 mg L^-1^, showing inhibitory effects. In G4, the POC values suddenly dropped to 75.4% and 51.4% at 0.1 and 10.0 mg L^-1^, respectively. The drops in POC values indicated significant inhibitory effects on initial reproduction in later generations. Meanwhile, the POC values of Cu, Pb and Zn did not show sudden drops, but showed continuous drops from G2 to G4. For example, the POC values of Pb at 10.0 mg L^-1^ decreased from 83.1% (G2) to 41.4% (G4). In G4, the initial reproduction suffered the greatest inhibition which followed an order of Pb > Cd > Cu > Zn (p < 0.05) at 10.0 mg L^-1^. Moreover, reproduction suffered more inhibitions than feeding (Fig 1) and growth (Fig 3).

### Effects of metals on antioxidants in nematode

**Fig 4:**
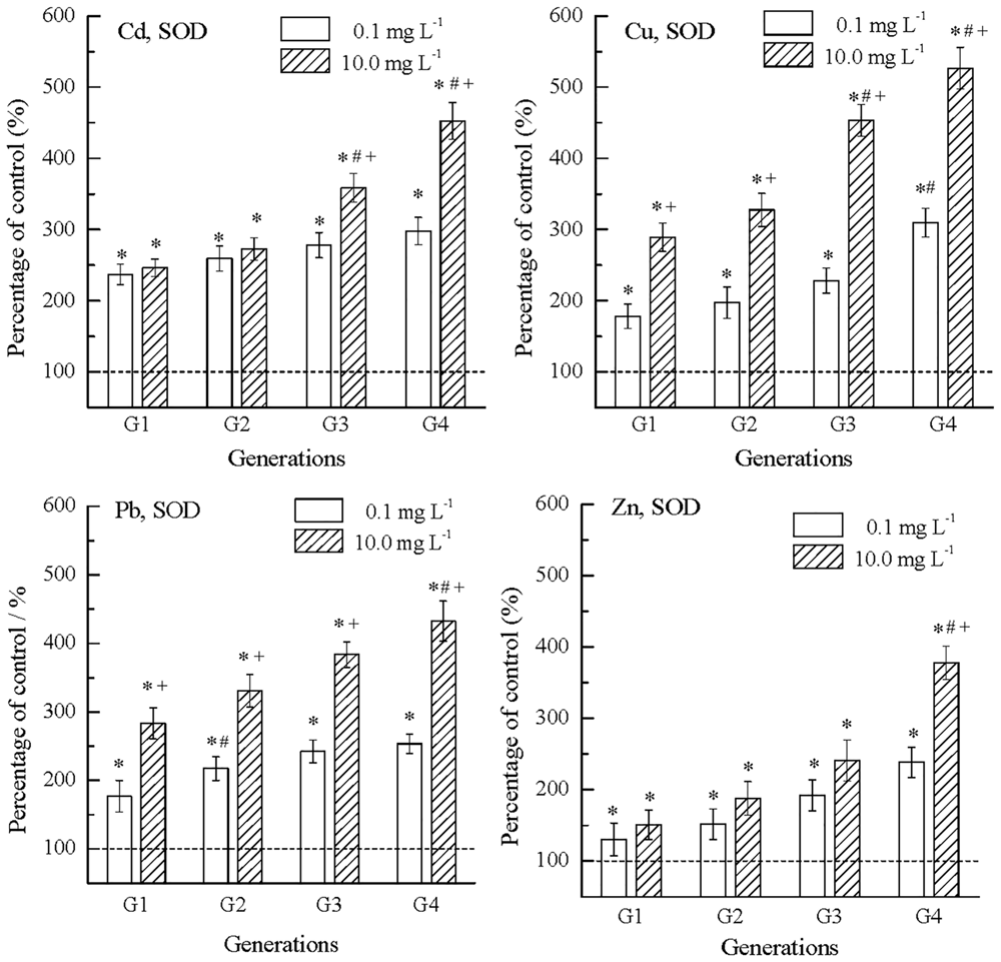
Superoxide dismutase (SOD) in *C*. *elegans* increased over generations from G1 to G4 in exposure to Cd, Cu, Pb and Zn. The average SOD of nematodes in the control was 1.2E-01 U/ng protein, and was normalized to 100% (dash line); *: significantly different from the concurrent control, p < 0.05; #: significantly from the earlier generation, p < 0.05; +: significantly different from the lower concentration, p < 0.05; Error bar: standard error.

Effects of metals on nematode SOD are shown in Fig 4. The POC values of all metals were higher than 100%, showing stimulatory effects. In Cd, when exposure generation increased from G1 to G4, the POC values at 0.1 mg L^-1^ increased from 237.1% to 298.0%, and those at 10.0 mg L^-1^ increased from 246.3% to 452.7%. That is to say, the stimulation levels increased over concentrations and generations. Moreover, such concentration- and generation-dependent increases in stimulation levels were also observed in Cu, Pb and Zn. The stimulation by Zn was the lowest among metals.

**Fig 5:**
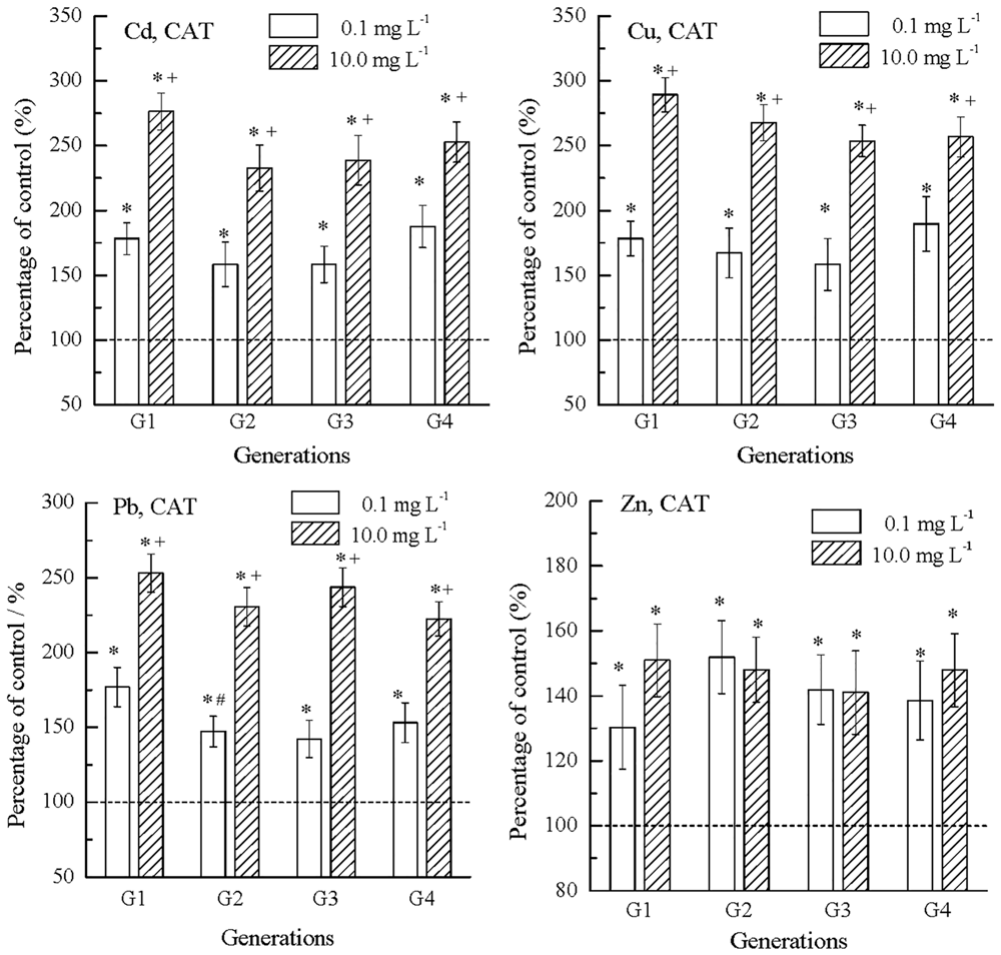
Catalase (CAT) in *C*. *elegans* showed stimulation in multigenerational exposure to Cd, Cu, Pb and Zn. The average CAT of nematodes in the control was 1.9E-04 U/ng protein, and was normalized to 100% (dash line); *: significantly different from the concurrent control, p < 0.05; #: significantly from the earlier generation, p < 0.05; +: significantly different from the lower concentration, p < 0.05; Error bar: standard error.

Effects of metals on nematode CAT are shown in Fig 5. Similar to the results of SOD, the POC values of all metals were higher than 100%, showing stimulatory effects. At 0.1 mg L^-1^, the POCs were 158.3% to 187.5%, 158.3% to 189.5% and 142.2% to 176.9% in Cd, Cu and Pb, respectively. At 10.0 mg L^-1^, the POCs were 232.6% to 276.3%, 253.6% to 289.3% and 222.4% to 253.1% in Cd, Cu and Pb, respectively. The POC values showed that the stimulation levels in CAT were greater at higher metal concentrations, which was similar to the concentration-dependent increases in SOD stimulation levels. Contrary to SOD, the stimulation levels of CAT in Cd, Cu and Pb were similar among generations (G1 to G4). Differently from results of other metals, the stimulation levels of CAT in Zn were the lowest among metals (p < 0.05), and they showed similar POCs (130.3% to 150.9%) among generations and between concentrations.

## Discussion

### Effects of metals on nematodes in the first generation

In the first generation (G1), the nematode feeding, growth and initial reproduction were not significantly influenced by metals, but SOD and CAT showed significant stimulatory effects. The effects in the present study are quite different from earlier reported effects at similar concentration levels. For example, in a 24 h exposure, 50% inhibitions on feeding were achieved at 14.4, 3.11 and 9.3 mg L^-1^ in Cd, Cu and Pb, respectively. At the same time, 50% inhibitions on growth were reached at 16.6, 3.6 and 8.5 mg L^-1^, respectively [8]. In a 72 h exposure, 50% inhibitions on reproduction were reached at 17.0, 2.0–2.5 and 6.2 mg L^-1^ in Cd, Cu and Pb, respectively [8, 9]. Nematodes avoided food spots containing 1–2 mg L^-1^ of Zn and Pb [12]. In addition, the CAT inductions were weaker than those in an earlier report where CAT was stimulated by 20 mg L^-1^ dietary Cu to a POC of nearly 10000% [11].

Such differences might be explained by the exposure arrangements. First, exposure in earlier studies started with adult nematodes (3 d after synchronization) and lasted 1 to 3 days, while exposure in the present study started with synchronized eggs and lasted 5 days. The weaker effects of metals in the present study indicated that younger nematodes had higher tolerance than older ones. The indication was consistent with the higher adaptabilities in young nematodes than old ones against oxidative stress [25]. Meanwhile, it was reported that L1 nematodes showed the greatest susceptibility among L1-L4 and adult nematodes in metal toxicities [26]. Second, the reproduction in the present study was the initial brood size in the earlier period of life, while earlier studies employed the whole brood size over the entire reproduction time. The present results demonstrated the delay or decrease of the initial reproduction, which might be compensated by later reproduction or longer reproduction duration [22]. The exact reasons for the observed differences needs further investigations.

### Effects in G1 and G2 with continuous exposure vs. those with one-generation exposure

In previous studies, EC_50_ values on body length in G1 were 10.77, 0.70, 0.83 and >24.85 mg L^-1^ in Cd, Cu, Pb and Zn, respectively. Meanwhile, their values in G2 without exposure were 0.30, 0.22, 0.12 and 4.25 mg L^-1^, respectively. The comparison of EC_50_ values indicated that Cd, Cu, Pb and Zn caused greater inhibitions on the nematode growth in G2 than in G1. Such greater inhibition in G2 was also observed in effects on nematode locomotion [1]. There were two differences between the results in the present study and those in the earlier ones. One is that nematodes in G2 showed similar effects to those in G1. The other one is that the effects at 0.1 and 10.0 mg L^-1^ in the present study were significantly lower than those in the earlier studies.

Such differences could be due to that the adverse effects were rescued by the presence of food. Food showed protective effects because it provided necessary energy for the development and the tissue repair [15, 27]. The presence of food in the multigenerational exposure might decrease the deterioration over generations and thus hindered significant effect changes between G1 and G2. However, food was demonstrated as a main toxicity pathway in Cu toxicities [11]. Therefore, the influence of food might be double-face and depended on other aspects in the experiment. For example, food might have different roles at different life stages, and further studies are needed for such explanation.

### Interesting effects in G3 to G4 under multigenerational challenges

In G3 and G4, SOD and CAT continued to show significant stimulatory effects. Meanwhile, feeding, growth and initial reproduction started to show significant inhibitions. The existence of both stimulatory and inhibitory effects indicated trade-off effects among indicators in G3 and G4. The trade-off effects were supported by the negative correlations between antioxidant and growth (or reproduction) (Pearson’s r greater than -0.74, S1 Table). Such trade-offs suggested the intrinsic energy balance among endpoints. That is to say, when the antioxidants were stimulated to scavenge the ROS generated by metals to keep maintenance [28], they diverted energy from growth and reproduction [29]. Compared with growth, the reproduction suffered more inhibitions, which can be explained by the higher energy demand. It was found that under exogenous oxidative insult, the nematode antioxidant defense (somatic maintenance) increased while the investment in reproduction decreased [13]. At the same time, the balance between antioxidant and growth was also observed in bacteria and birds [30–32]. Therefore, the trade-offs among indicators widely exist. To the best of our knowledge, the present study showed multigenerational trade-offs for the first time.

Heavy metals are well known for their abilities of metals to provoke ROS and subsequent oxidative stress and DNA damage [33]. The increases of antioxidants in nematodes over generations in the present study indicated some adaptation abilities of the nematodes against the metal toxicities. Such adaptation was also observed in multigenerational effects of Cd on *Spodoptera exigua* which showed lower mortality in later generations [5]. The adaptation might come from increases of inner metal concentrations in the organisms. In copepod and insect, it was found that metal accumulation increased over exposure generations [4, 5]. In nematodes, quantum dots [34], graphite nanoplatelets [35], and fluorescent nanodiamonds [36] were reported to transit from the alimentary system to the reproductive system, and even from the parent to the embryos and into the hatched larvae in the offspring. Yet, it needs further investigation to find whether the antioxidant increases over generations in the present study were accompanied with metal accumulation.

Notably, lead (Pb) is a well-known toxic pollutant and able to provoke oxidative stress and antioxidant responses at mg L^-1^ [37]. Meanwhile, Cu and Zn serve as micronutrients, and they were at mg L^-1^ in human blood [38] and mg kg^-1^ in flour and corn [39]. Such difference explained the results in the present study where Pb caused the most severe inhibition on reproduction and significantly stimulated antioxidant responses, while Zn showed the lowest antioxidant stimulation and Cu slightly stimulated feeding at 10.0 mg L^-1^ in G1. The different effects of metals were also reported in earlier studies where metallothionein mutant (*mtl*) nematodes were more sensitive to Cu than Pb, Cd and Zn [40]. Yet, the exact mechanisms for such differences among metal effects need further investigations.

Another noteworthy point was that the exposure concentrations in the present study were environmentally relevant [16], while environmental effects at such concentrations were seldom reported. The multigenerational effects in the present study, especially the latent responses of feeding, growth and reproduction in G3 and G4, indicated that long-term influences of metal pollutants would be underestimated if the multigenerational challenges were not considered. Our finding urged more multigenerational studies in judging the long-term effects of environmental pollutants in future studies.

## Conclusion

In G1 and G2, the nematode feeding, growth and initial reproduction were not significantly influenced by the chosen metals, while SOD and CAT showed significant stimulatory effects. In G3 and G4, the feeding, growth and initial reproduction showed apparent inhibitory effects, while SOD and CAT were continuously stimulated, and the stimulatory effects on SOD increased from G1 to G4. The significantly inhibitory effects on reproduction and interesting trade-offs among indicators were not observed until in later generations, demonstrating the importance of multigenerational challenges in judging the long-term influences of environmental pollutants.

## Supporting Information

S1 Table Pearson’s correlation coefficient (Pearson’s r) among feeding, growth, reproduction and superoxide dismutase (SOD) in *C*. *elegans* after multigenerational exposure to metals. *: p < 0.1; **: p< 0.05; ***: p < 0.01.
(DOCX)
