# Supplementary material for: Multigenerational Effects of Heavy Metals on Feeding, Growth, Initial Reproduction and Antioxidants in Caenorhabditis elegans
Source: PLoS One. 2016 Apr 26;11(4):e0154529. doi: 10.1371/journal.pone.0154529 (PMC4846010; doi:10.1371/journal.pone.0154529)
Supplement: S1 Table — *: p < 0.1; **: p< 0.05; ***: p < 0.01. (DOCX) [file pone.0154529.s001.docx]

**S1 Table. Pearson’s correlation coefficient (Pearson’s r) among feeding, growth, reproduction and superoxide dismutase (SOD) in *C. elegans* after multigenerational exposure to metals.** *: p < 0.1; **: p< 0.05; ***: p < 0.01.

|  |  |  | Feeding | Growth | Reproduction |
| --- | --- | --- | --- | --- | --- |
| Cd | 0.1 mg/L | Growth | 0.97*** | - | - |
|  |  | Reproduction | 0.67 | 0.68 | - |
|  |  | SOD | -0.74 | -0.81 | -0.87 |
|  | 10.0 mg/L | Growth | 0.98*** | - | - |
|  |  | Reproduction | 0.74 | 0.76 | - |
|  |  | SOD | -0.79 | -0.85 | -0.95** |
| Cu | 0.1 mg/L | Growth | 0.99*** | - | - |
|  |  | Reproduction | 0.77 | 0.83* | - |
|  |  | SOD | -0.40 | -0.50 | -0.79 |
|  | 10.0 mg/L | Growth | 0.97*** | - | - |
|  |  | Reproduction | 0.84* | 0.93** | - |
|  |  | SOD | -0.89* | -0.90* | -0.88* |
| Pb | 0.1 mg/L | Growth | 0.97*** | - | - |
|  |  | Reproduction | 0.88** | 0.91** | - |
|  |  | SOD | -0.80 | -0.84 | -0.98** |
|  | 10.0 mg/L | Growth | 0.98*** | - | - |
|  |  | Reproduction | 0.88** | 0.90** | - |
|  |  | SOD | -0.80 | -0.83 | -0.99** |
| Zn | 0.1 mg/L | Growth | 0.97*** | - | - |
|  |  | Reproduction | 0.84* | 0.84* | - |
|  |  | SOD | -0.70 | -0.80 | -0.98** |
|  | 10.0 mg/L | Growth | 0.98*** | - | - |
|  |  | Reproduction | 0.83* | 0.81* | - |
|  |  | SOD | -0.67 | -0.74 | -0.96** |
